# Nonalcoholic Fatty Liver Disease in University Rugby Football Players

**DOI:** 10.3389/fendo.2018.00341

**Published:** 2018-06-20

**Authors:** Shinsuke Nirengi, Mami Fujibayashi, Sachiko Furuno, Akihiko Uchibe, Yasuharu Kawase, Shin Sukino, Yaeko Kawaguchi, Satomi Minato, Kazuhiko Kotani, Naoki Sakane

**Affiliations:** ^1^Clinical Research Institute, Division of Preventive Medicine, National Hospital Organization Kyoto Medical Center, Kyoto, Japan; ^2^Division of Physical and Health Education, Setsunan University, Neyagawa, Japan; ^3^Josho Welfare Co., Ltd., Osaka, Japan; ^4^Graduate School of Human Science and Environment, University of Hyogo, Himeji, Japan; ^5^Division of Community and Family Medicine, Jichi Medical University, Shimotsuke, Japan

**Keywords:** NAFLD, rugby football, athletes, diet, BMI

## Abstract

Physical activity improves various metabolic disturbances. The effect of physical activity on non-alcoholic fatty liver disease (NAFLD) has not been defined, particularly in athletes who are able to consume a diet to increase body mass. The aim of this study was to evaluate the prevalence of NAFLD and associated factors of NAFLD among male university rugby football players [*n* = 69, 37 forwards (FW) and 32 backs (BK)], relative to age-matched controls (CON; *n* = 29). For FW players exercise consists of physical contact play, such as ruck, mall, scrum, and tackle. For BK players exercise consists of sprints and endurance running. Liver function tests and bioimpedance analysis to assess body composition were performed. Subjects consuming ≤ 20 g/day of ethanol and exhibiting an aspartate transaminase (AST) level ≥ 33 U/L, and/or alanine transaminase (ALT) level ≥ 43 U/L, were considered to have NAFLD. The *PNPLA3* and *MTP* genotypes were determined using real-time polymerase chain reaction (PCR). The body mass index, body fat mass, and lean body mass were significantly higher in the FW group than in the BK and CON groups (*P* < 0.05). The total cholesterol, low-density lipoprotein cholesterol, triglyceride, AST, ALT, and alkaline phosphatase levels were significantly higher in the FW group than in the CON group (*P* < 0.05). The prevalence of NAFLD was significantly higher in the FW group than in the BK group and CON group (18.9, 8.6, and 0.0%, respectively), whereas there were non-significant between-group differences in the frequency of the *PNPLA3* and *MTP* genotypes. These findings indicate that rugby football players, especially those in the FW position, are at higher risk of developing NAFLD, which emphasizes the role of diet and exercise in the development of NAFLD.

## Introduction

Lifestyle-related metabolic diseases associated with obesity are rising worldwide. Moreover, obesity is strongly related to the development of nonalcoholic fatty liver disease (NAFLD) in both pediatric and adult populations (1, 2). NAFLD is characterized by an excess accumulation of lipids in the liver (>5–10% of the weight of the liver) which is not due to excess alcohol consumption or steatosis (3, 4). NAFLD is an important cause of lifestyle-related metabolic diseases (5, 6). As well, the development of nonalcoholic steatohepatitis increases the risk of liver cancer and cardiovascular-related mortality (7, 8). Thus, to lower the risk of NAFLD, strategies to understand its development are warranted (9).

Risk factors for NAFLD include age, body mass index (BMI), and sex, as well as lifestyle factors (i.e., diet and exercise) and genetic factors (10–16). Exercise is generally identified as a strategy to improve NAFLD, independent of weight loss (12). The prevalence of NAFLD would be thus expected to be lower among athletes. However, athletes can consume a heavy diet to increase (or maintain) a larger body mass. As an athletic example, rugby football is a popular team contact sport (17–19). A rugby team consists of 13 players, six forwards (FW) and seven backs (BK), with FWs typically having a larger body mass than BKs. The pattern of physical exercise differs between the positions of FW and BK, as short-duration anaerobic activity (ruck, mall, scrum, and tackling) is needed at FW and sprint and endurance running is needed at BK. The dietary pattern required for position-specific body compositions is also likely to differ between these positions.

Notably, the investigation of NAFLD in relation to diets accompanied by full exercise may be useful to understand the development of NAFLD. The aim of our study was to evaluate the prevalence of NAFLD among rugby football players to determine whether there are position-related factors that influence the onset of NAFLD.

## Material and methods

### Participants

Sixty-nine male university rugby players (37 FW and 32 BK) were enrolled in this study. All players were with the A-League, the highest level of the local league in Japan, practicing 6 days per week, 3 h per day. We also recruited 29 controls (CON) from the same university by advertising on posters; these subjects did not engage in regular exercise (Table 1). Prospective participants who habitually consumed alcohol (≥20 g/day) or had a prior history of myocardial injury [defined as a creatinine phosphokinase level >3X the upper reference limit (≥ 600 IU/L)] and chronic viral liver disease such as hepatitis B or C, were excluded. This study was carried out in accordance with the recommendations of principles of the Declaration of Helsinki (Fortaleza 2013). The study protocol was approved by the Ethics Committee of the Institutional Review Board of Setsunan University (approval number: 2013–005), and all participants provided informed consent.

**Table 1:** Demographic, biochemical, clinical characteristics of rugby football players.

**Variables**	**FW** **(*n* = 37)**	**BK** **(*n* = 32)**	**CON** **(*n* = 28)**
Age, year	19.5 (19.0–20.3)^†^	20.0 (19.0–21.0)	20.0 (20.0–22.0)
Height, cm	178.0 (173.1–181.4)^※^^†^	170.5 (166.6–175.8)	171.0 (169.3–175.0)
Weight, kg	92.7 (86.5–103.0)^※^^†^	74.0 (68.9–81.1)^†^	63.0 (57.5–70.8)
BMI, kg/m^2^	29.1 (27.3–32.8)^※^^†^	25.3 (23.4–26.9)^†^	21.5 (20.0–24.0)
WC, cm	96.3 (84.2–112.8)^※^^†^	84.4 (78.9–89.5)^†^	73.5 (68.3–78.8)
Body fat, %	21.6 (16.1–28.5)^※^^†^	15.2 (10.6–19.7)^†^	14.0 (11.3–18.0)
Lean body mass, kg	72.2 (69.1–77.5)^※^^†^	63.9 (57.6–66.8)^†^	54.5 (49.8–59.0)
HbA1c, %	5.2 (5.0–5.2)	5.1 (5.0–5.4)	5.2 (5.0–5.3)
TC, mmol/L	4.9 (4.3–5.3)^†^	4.4 (4.0–4.7)	4.3 (3.8–4.7)
HDL-C, mmol/L	1.4 (1.3–1.6)	1.6 (1.4–1.7)	1.6 (1.3–1.7)
LDL-C, mmol/L	2.9 (2.4–3.4)^†^	2.6 (2.2–2.7)^†^	2.5 (2.0–2.9)
TG, mmol/L	1.6 (1.2–2.7)^†^	1.3 (1.0–1.8)	1.0 (0.6–1.4)
AST, IU/L	22.5 (19–26.3)^†^	21.0 (19.0–24.0)	18.0 (15.0–22.5)
ALT, IU/L	21.5 (17.8–36.5)^†^	19.0 (13.5–28.5)	17.0 (13.3–24.0)
ALP, IU/L	298 (17.8–36.5)^†^	300 (238.5–340.5)^†^	232.5 (197.5–264.3)
LDH, IU/L	183.0 (161.5–199.5)	179 (159.5–188.5)	177 (159.3–198.8)
γ-GT, IU/L	24.5 (19.5–32.5)^†^	20.0 (17.0–24.5)	19.0 (16.0–23.8)
CPK, IU/L	149.5 (108.5–201.5)	143.0 (103.5–189.5)	132.0 (103.5–219.3)
CHE, IU/L	371.0 (308.5–430.5)	337.0 (304.5–378.5)	331.5 (288.3–361.5)
UA, umol/L	359.9 (319.7–416.4)	339.0 (300.4–380.7)	380.7 (316.7–398.5)
Adiponectin,	7.6 (6.1–9.4)	7.1 (5.3–9.0)	–
Alcohol intake, g/day	0.0 (0.0–5.6)	2.2 (0.0–6.2)	3.0 (0.0–7.8)
Smoking habit, %	16.2	18.8	14.3
*PNPLA3* (CC/CG/GG), %	37.8/48.6/13.5	40.6/50.0/9.4	28.6/57.1/14.3
*MTP* (GG/GT/TT), %	64.9/35.1/0.0	71.9/28.1/0.0	75.0/25.0/0.0

FW, forwards; BK, backs; CON, control; WC, waist circumference; TC, total cholesterol; HDL, high-density lipoprotein; LDL, low-density lipoprotein; TG, triglyceride; AST, aspartate amino transferase; ALT, alanine amino transferase; ALP, alkaline phosphatase; LDH, lactate dehydrogenase; γ-GT, gamma-glutamyl transpeptidase; CPK, creatine phosphokinase; CHE, cholinesterase; UA, uric acid; PNPLA3, patatin-like phospholipase domain-containing protein 3; MTP; microsomal triacylglyceride-transfer protein

※ P < 0.05 vs. BK,

† *P < 0.05 vs. CON*.

Body weight and body fat content were measured using a body fat analyzer (Inbody 430, Biospace, Seoul, Korea). Waist circumference (WC) was measured midway between the lowest rib margin and the iliac crest. BMI was calculated using measured weight and height (kg/m^2^). Handgrip strength of the dominant hand was measured using a TKK 5401 grip dynamometer (Takei Kiki Kogyo, Tokyo, Japan); three trials were completed and the largest value was used for analysis. The fastest 1 km time trial record obtained during club activities was used for analysis. Daily intake of energy and nutrients during the preceding month were measured brief-type self-administered diet history questionnaire (BDHQ) (20), as well as smoking habits, were assessed using a validated brief self-administered questionnaire.

### Blood analysis

Blood glucose concentrations were measured using the hexokinase method (Serotec Co., Ltd., Hokkaido, Japan). Total cholesterol (TC), high-density lipoprotein (HDL)-C, low-density lipoprotein (LDL)-C, triglycerides (TG), and uric acid (UA) levels were determined by enzymatic methods (Kyowa Medex Co., Ltd., Tokyo, Japan) using the automatic biochemical analyzer (JCA-BM8060; JEOL, Ltd., Tokyo, Japan). AST, alanine amino transferase (ALT), alkaline phosphatase (ALP), lactate dehydrogenase (LDH), gamma-glutamyl transpeptidase (γ-GT), creatine phosphokinase (CPK), and cholinesterase (CHE) levels were measured using commercial assay kits (CicaLiquid, KANTO CHEMICAL CO., INC., Tokyo, Japan). Serum adiponectin levels were measured using an enzyme-linked immunosorbent assay kit (Otsuka Pharmaceutical Co., Ltd., Tokyo, Japan), with these measures obtained only in the FW and BK groups. The NAFLD is defined on the basis of the serum level of AST level ≥ 33 U/L, and/or ALT level ≥ 43 U/L as a surrogate maker for NAFLD (6, 21, 22).

### Genotyping

Genomic DNA was extracted from venous blood (2.0 μl) from each participant using a DNA Extract All Reagents kit (Applied Biosystems, Yokohama, Japan), according to the manufacturer's instructions. After extraction, the genomic DNA was immediately stored at −30°. Genotyping was performed for patatin-like phospholipase domain-containing 3 (*PNPLA3*: rs738409) and microsomal triacylglyceride-transfer protein (*MTP*: rs180084), using an ABI prism 7300 (Applied Biosystems, Yokohama, Japan). *PNPLA3* and *MTP* expression has previously been shown to be strongly associated with liver fat content (14–16). In brief, 5 μl GTXpress Master Mix (Applied Biosystems, Yokohama, Japan), 0.5 μl SNP-specific TaqMan genotyping assay mix (Applied Biosystems, Yokohama, Japan), 2.5 μl nuclease-free H_2_O, and a 2.0 μl DNA solution were added per well. The denaturation began at 95°C for 20 s, with 40 cycles of incubation at 95°C for 15 s then annealing and extension at 60°C for 1 min. The allele frequencies of SNP genotypes were tested for the Hardy-Weinberg equilibrium.

### Statistical analysis

Data were expressed as median (25–75th percentile). One-way analysis of variance (ANOVA) with Scheffe's post hoc test (in parametric analysis) or Mann-Whitney U test with Kruskal–Wallis test (in nonparametric analysis) were performed to determine differences among the three groups (FW, BK, and CON). Differences between FW and BK were analyzed using a Student's *t*-test. Categorical variables including smoking habit, genotypes and prevalence of NAFLD, were compared using the Chi-square (χ2) test. A multivariate regression analysis was performed to evaluate the relationship between the the NAFLD and other measurements such as age, BMI, smoking, energy intake, TG, adiponectin and genotypes. Values were considered statistically significant at *P* < 0.05. All statistical analyses were performed using SPSS (IBM Corp., SPSS Statistics 20.0, Armonk, NY, U.S.A.).

## Results

The clinical characteristics for the FW, BK and CON groups are summarized in Table 1. The BMI, body fat mass and lean body mass were significantly higher in the FW group than in the BK and CON groups (*P* < 0.05). The TC, LDL-C, TG, AST, ALT, γ-GT, and ALP levels were significantly higher in the FW group than in the CON group (*P* < 0.05). Handgrip strength tended to be higher in the FW group [473.3 (453.0–501.0) N], relative to the BK group [452.8 (405.2–501.8) N] (*P* = 0.09). The 1 km time trial was significantly slower in the FW group [237.5 (224.0–259.0) s], compared with the BK group [218.0 (211.5–222.8) s] (*P* < 0.05).

**Figure 1:**
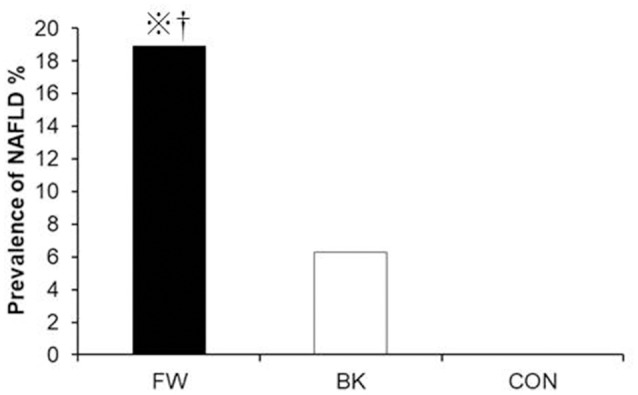
Prevalence of non-alcoholic fatty liver disease. The prevalence of NAFLD was compared using the chi-squared (χ2) test.*P* < 0.05 vs. BK, ^※^*P* < 0.05 vs. CON.

With regard to diet, energy intake was higher in the FW (2560 ± 772 kcal/day) and BK groups (2492 ± 560 kcal/day) than in the CON group (1902 ± 691 kcal/day); all other dietary parameters were comparable among the groups. Importantly, despite, either both FW and BK had habitual vigorous exercise, the prevalence of NAFLD evaluated by AST and/or ALT was significantly higher in the FW group than in either the BK or CON group (18.9, 6.3, and 0%, respectively) (Figure 1).

There were no significant differences in *PNPLA3* and *MTP* genotype frequencies among the groups. The genotypic distributions of *PNPLA3* (χ^2^ = 1.7*, P* = 0.41) and *MTP* (χ^2^ = 3.6, *P* = 0.17) were in accordance with the Hardy-Weinberg equilibrium, with frequencies similar to previously reported values for the Japanese population (16, 23).

On multivariate regression analysis, NAFLD was significantly and independently correlated with BMI (Standardized β = 0.32, *P* < 0.05), energy intake (Standardized β = 0.31, *P* < 0.05), TG level (Standardized β = 0.25, *P* < 0.05) and adiponectin level (Standardized β = −0.23, *P* < 0.05) (Table 2). In a sub-analysis restricted to the FW group, NAFLD was significantly and independently correlated with BMI (Standardized β = 0.45, *P* < 0.05) and energy intake (Standardized β = 0.34, *P* < 0.05).

**Table 2:** Multiple regresssion analysis for NAFLD in FW and BK.

**Variable**	**β**	**Standardized β**	***P***
Age	−0.01	−0.02	0.87
BMI	0.03	0.32	<0.05
Smoking	0.05	0.06	0.60
Energy intake	0.000159	0.31	<0.05
TG	0.09	0.25	<0.05
Adiponectin	−0.03	−0.23	<0.05
*PNPLA3*	−0.04	−0.08	0.43
*MTP*	0.02	0.03	0.78

*PNPLA3, patatin-like phospholipase domain-containing protein 3; MTP, microsomal triacylglyceride-transfer protein*.

## Discussion

The main finding of this study was that the prevalence of NAFLD in the FW group was higher than those in the BK and CON groups. Further, BMI, TG, and adiponectin levels, as well as energy intake, were all identified as independent factors associated with NAFLD. Our findings may be surprising, as physical activity is known to confer a protective effect against non-communicable diseases (24), including NAFLD. *A priori*, we had anticipated that the physical exercise regime of the players enrolled in our study (6 days/week × 3 h/day) would have been sufficient to offer protection against NAFLD. Although we failed to evaluate physical activity in these subjects, FW had higher physical activity than BK [2812.0 (1670.0–2511.0] vs. 1662.5 [1447.3–2173.0] kcal/day) in 80 juniors of the same team, according to a questionnaire. FW were reported to have a higher number of contacts (1.5–2.0 times) in previous studies (25, 26). As collision-based activities have a high energy cost (27), a previous study also showed that FW had higher energy expenditure than BK, although FW reported a lower total distance (26, 28). Although the FW might report more activity, their prevalence of NAFLD is high; this may affect energy intake and body fat mass. However, total energy intake was similar between the FW and BK groups on BDHQ. Notably, BDHQ showed satisfactory ranking ability in the same group (20). However, BDHQ might be biased; therefore, the energy intake of rugby football players in this study was comparatively lower than that reported in a previous study (18). Currently, there is no accurate nutrition questionnaire for athletes because of the complex influence of sport-specific factors, such as periodized training, large portion sizes, and widespread use of rapidly evolving sports foods and supplements (29). However, a registered dietitian provided breakfast, lunch, dinner and additional nutrition for some portions of each day. The respective total energy, protein, fat, and carbohydrate intakes in the FW and BK groups were ~4880 vs. 3,920 kcal/day, 174 vs. 156 g/day, 138 vs. 135 g/day, and 735 vs. 520 g/day. This athlete-NAFLD model might support the concept of the development of NAFLD due to an imbalance between diet and exercise.

Generally, the lifestyle-related diseases, including obesity and metabolic syndrome, are a cause of NAFLD (30, 31). Our study supported this general idea (30, 31); accordingly, excess energy intake, BMI, TG, and/or adiponectin [an adipocyte-driven hormone (30, 32–34)], which is increased in metabolic disorders related to lifestyle) were associated with the presence of NAFLD. Notably, excess energy intake and BMI were the influential parameters in the FW group. A previous study reported that energy restriction with weight reduction improved the symptoms of NAFLD (35). One of the mechanisms of excess energy intake associated with the development of NAFLD is a decrease in the liver energy sensor, 5′-AMP activated protein kinase (AMPK) (30). Suppression of AMPK activation decreases lipolysis and fatty acid oxidation through an activation of the adipocyte triglyceride lipase (ATGL) and carnitine palmitoyltransferase-1 (CPT-1) pathway; additionally, it increases lipogenesis through a downregulation of the acetyl-CoA carboxylase (ACC) pathway (36).

Rugby football is a contact sport and greater body mass is associated with a superior performance. The results of our study were consistent with previous findings of higher body fat among FWs, relative to BKs (17, 18, 37–39). Increases in body fat mass negatively correlate with power and velocity (17), as well as running endurance, which are important performance parameters for an 80-min rugby football game (played as two 40-min halves). In our cohort of rugby football players, those with a higher BMI had a slower 1 km time, indicative of a decrease in physical endurance capacity, with excess body fat (data not shown). A recent study reported that the hepatokine selenoprotein P, which is secreted in cases of NAFLD, causes aerobic exercise resistance (40). In contrast, fat-free mass is the parameter that best predicts the likelihood of being classified as an International or National player, highlighting the importance of muscle mass as a performance indicator in this sport (37). However, it is difficult to increase muscle mass without a concomitant increase in body fat mass; this increase in fat mass decreases exercise performance (38) and increases the risk for NAFLD. Thus, it is critical to consider the necessary degree of balance between health and sport performance for athletes who must increase body mass.

The combination of diet and exercise treatment improves the pathogenesis of NAFLD as a general consensus (41, 42). A 9-month study, using treatment with a diet and moderate exercise, revealed a 35.2% reduction in liver fat and a significant change in hepatic enzyme levels, resulting NAFLD resolution in 40% of subjects (41). Subjects with NAFLD have generally been advised to lose 5–10% of their total body weight, via diet, and regular exercise (42). However, the ideal ratio of this combination [e.g., which treatment (diet or exercise) is superior to improve the NAFLD] remains to be determined in the real world. The ratio may also differ in various situations. Interestingly, our study showed that even the rugby football players who had a full exercise regimen developed NAFLD. This might suggest that, besides weight management, diet is more influential than exercise in the onset of NAFLD among rugby football players (especially those in a FW position); hence, the balance between diet and exercise is a key aspect in the development of NAFLD. The optimal balance of diet and exercise to prevent NAFLD might be determined for athletes. Further studies are needed to clarify the molecular mechanisms of the onset of NAFLD, as well as to determine an optimal program for the prevention of NAFLD in athletes.

There are limitations to this study that may affect our interpretation of the results. First, as this study was based on field research, liver biopsy methods, proton magnetic resonance spectroscopy methods (43, 44), and abdominal ultrasound methods (45) were not used to assess the NAFLD. Although we attempted to evaluate the fatty liver index (46, 47) and hepatic steatosis index (48), NAFLD was excluded in 26% and >50%, respectively, which might indicate over-evaluation because the AST might be affected by exercise; moreover, BMI and WC were extremely high (49) because rugby football players have a large amount of muscle. Second, the sample size was relatively small and was drawn from a single institution. Third, the causality of the results remains unclear, as this was a simple observational study. Fourth, we did not evaluate exercise activity and failed to estimate total energy intake by using BDHQ. The doubly labeled water method and 3-day food intake record method would be needed to investigate detailed energy and lifestyle balances. Fifth, the FW group had slightly higher CPK levels than did the BK or CON group; therefore, the prevalence of NAFLD in FW may have been overestimated. Future studies are needed to validate our findings.

## Conclusion

The findings of our study indicate that even university athletes, including rugby football players (especially those in a FW position), may be at risk for NAFLD, despite their regularly high levels of physical exercise. We must further investigate the molecular mechanisms of NAFLD, as well as the effects of dietary interventions on NAFLD.

## Author contributions

SN, MF, and NS designed the study. SN, MF, SF, AU, and YK collected and assembled of data. SN and NS performed the statistical analysis and prepared the manuscript. KA, SS, YK, SM, and KK did the trial management and helped to draft the manuscript with its critical review and. All authors are in agreement with the manuscript and declare that the content has not been published elsewhere.

### Conflict of interest statement

The authors declare that the research was conducted in the absence of any commercial or financial relationships that could be construed as a potential conflict of interest. The handling Editor declared a shared affiliation though no other collaboration with one of the authors KK.

## References

[1] Lavine JE Schwimmer JB Van Natta ML Molleston JP Murray KF Rosenthal P. Nonalcoholic steatohepatitis clinical research network. effect of vitamin E or metformin for treatment of nonalcoholic fatty liver disease in children and adolescents: the TONIC randomized controlled trial. JAMA (2011) 305:1659–68. 10.1001/jama.2011.520 21521847 PMC3110082

[2] Nobili V Alkhouri N Alisi A Della Corte C Fitzpatrick E Raponi M. Nonalcoholic fatty liver disease: a challenge for pediatricians. JAMA Pediatr. (2015) 169:170–6. 10.1001/jamapediatrics.2014.2702 25506780

[3] Rinella ME. Nonalcoholic fatty liver disease: a systematic review. JAMA (2015) 313:2263–73. 10.1001/jama.2015.5370 26057287

[4] Neuschwander-Tetri BA Caldwell SH. Nonalcoholic steatohepatitis: summary of an AASLD single topic conference. Hepatology (2003) 37:1202–19, 10.1053/jhep.2003.50193 12717402

[5] Newton K.P Hou J Crimmins N.A Lavine J.E Barlow S.E Xanthakos S.A. Nonalcoholic steatohepatitis clinical research network. Prevalence of prediabetes and type 2 diabetes in children with nonalcoholic fatty liver disease. JAMA Pediatr. (2016) 170:e161971 10.1001/jamapediatrics.2016.1971 27478956 PMC5479314

[6] Kotronen A Yki-Järvinen H Männistö S Saarikoski L Korpi-Hyövälti E Oksa H. Non-alcoholic and alcoholic fatty liver disease - two diseases of affluence associated with the metabolic syndrome and type 2 diabetes: the FIN-D2D survey. BMC Public Health (2010) 10:237. 10.1186/1471-2458-10-237 20459722 PMC2873937

[7] Fracanzani AL Pisano G Consonni D Tiraboschi S Baragetti A Bertelli C. Epicardial Adipose Tissue (EAT) Thickness is associated with cardiovascular and liver damage in nonalcoholic fatty liver disease. PLoS ONE (2016) 11:e0162473. 10.1371/journal.pone.0162473.eCollection2016 27627804 PMC5023162

[8] Fargion S Porzio M Fracanzani AL. Nonalcoholic fatty liver disease and vascular disease: state-of-the-art. World J Gastroenterol. (2014) 20:13306–24. 10.3748/wjg.v20.i37.13306 25309067 PMC4188888

[9] Michelotti GA Machado MV Diehl AM. NAFLD, NASH and liver cancer. Nat Rev Gastroenterol Hepatol. (2013) 10:656–65. 10.1038/nrgastro.2013.183 24080776

[10] Eguchi Y Hyogo H Ono M Mizuta T Ono N Fujimoto K. Prevalence and associated metabolic factors of nonalcoholic fatty liver disease in the general population from 2009 to 2010 in Japan: a multicenter large retrospective study. J Gastroenterol. (2012) 47:586–95. 10.1007/s00535-012-0533-z 22328022

[11] Eslamparast T Tandon P Raman M. Dietary composition independent of weight loss in the management of non-alcoholic fatty liver disease. Nutrients (2017) 9:E800. 10.3390/nu9080800 28933748 PMC5579594

[12] Houghton D Thoma C Hallsworth K Cassidy S Hardy T Burt AD. Exercise reduces liver lipids and visceral adiposity in patients with nonalcoholic steatohepatitis in a randomized controlled trial. Clin Gastroenterol Hepatol. (2017) 15:96–102.e3. 10.1016/j.cgh.2016.07.031 27521509 PMC5196006

[13] Winn NC Liu Y Rector RS Parks EJ Ibdah JA Kanaley JA Energy-matched moderate and high intensity exercise training improves nonalcoholic fatty liver disease risk independent of changes in body mass or abdominal adiposity - A randomized trial. Metabolism (2018) 78:128–40. 10.1016/j.metabol.2017.08.012 28941598

[14] Romeo S Kozlitina J Xing C Pertsemlidis A Cox D Pennacchio LA. Genetic variation in PNPLA3 confers susceptibility to nonalcoholic fatty liver disease. Nat Genet. (2008) 40:1461–5. 10.1038/ng.257 18820647 PMC2597056

[15] Zheng W Wang L Su X Hu XF. MTP−493G>T polymorphism and susceptibility to nonalcoholic fatty liver disease: a meta-analysis. DNA Cell Biol. (2014) 33:361–9. 10.1089/dna.2013.2238 24588800

[16] Hotta K Yoneda M Hyogo H Ochi H Mizusawa S Ueno T. Association of the rs738409 polymorphism in PNPLA3 with liver damage and the development of nonalcoholic fatty liver disease. BMC Med Genet. (2010) 11:172. 10.1186/1471-2350-11-172 21176169 PMC3018434

[17] Bell W Colley JP Evans WD Darlington SE Cooper SM. ACTN3 genotypes of Rugby Union players: distribution, power output and body composition. Ann Hum Biol. (2012) 39:19–27. 10.3109/03014460.2011.632648 22117592

[18] Imamura H Iide K Yoshimura Y Kumagai K Oshikata R Miyahara K. Nutrient intake, serum lipids and iron status of colligiate rugby players. J Int Soc Sports Nutr. (2013) 10:9. 10.1186/1550-2783-10-9 23402535 PMC3577441

[19] Nirengi S Fujibayashi M Tsuzaki K Furuno S Uchibe A Kawase Y. ACTN3 gene R577X polymorphism associated with high-density lipoprotein cholesterol and adiponectin in rugby players. Endocr Pract. (2016) 22:786–90. 10.4158/EP15963.OR 26919654

[20] Kobayashi S Honda S Murakami K Sasaki S Okubo H Hirota N. Both comprehensive and brief self-administered diet history questionnaires satisfactorily rank nutrient intakes in Japanese adults. J Epidemiol. (2012) 22:151–9. 10.2188/jea.JE20110075 22343326 PMC3798594

[21] Ruhl CE Everhart JE. Determinants of the association of overweight with elevated serum alanine aminotransferase activity in the United States. Gastroenterology (2003) 124:71–9. 10.1053/gast.2003.50004 12512031

[22] Yu AS Keeffe EB. Elevated AST or ALT to nonalcoholic fatty liver disease: accurate predictor of disease prevalence?. Am J Gastroenterol. (2003) 98:955–6. 10.1111/j.1572-0241.2003.07485.x 12809814

[23] Motohashi K Maruyama T Nakano S Maruyama C Kyotani S Saruta S [Title in Japanese]. J Japan Mibyou Syst Assoc. (2003) 9:258–60. 10.11288/mibyou1998.9.258

[24] Beaglehole R Bonita R Horton R Adams C Alleyne G Asaria P. Priority actions for the non-communicable disease crisis. Lancet (2011) 377:1438–47. 10.1016/S0140-6736(11)60393-0 21474174

[25] Roberts SP Trewartha G Higgitt RJ El-Abd J Stokes KA The physical demands of elite English rugby union. J Sports Sci. (2008) 6:825-833. 10.1080/02640410801942122 18569548

[26] Bradley WJ Cavanagh B Douglas W Donovan TF Twist C Morton JP. Energy intake and expenditure assessed ‘in-season’ in an elite European rugby union squad. Eur J Sport Sci. (2015) 15:469–79. 10.1080/17461391.2015.1042528 26055695

[27] Highton J Mullen T Norris J Oxendale C Twist C. The unsuitability of energy expenditure derived from microtechnology for assessing internal load in collision-based activities. Int J Sports Physiol Perform. (2017) 12:264–7. 10.1123/ijspp.2016-0069 27193085

[28] Morehen JC Bradley WJ Clarke J Twist C Hambly C Speakman JR. The assessment of total energy expenditure during a 14-day in-season period of professional rugby league players using the doubly labelled water method. Int J Sport Nutr Exerc Metab. (2016) 26:464–72. 10.1123/ijsnem.2015-0335 27096279

[29] Capling L Beck KL Gifford JA Slater G Flood VM O'Connor H. Validity of dietary assessment in athletes: a systematic review. Nutrient (2017) 9:E1313. 10.3390/nu9121313 29207495 PMC5748763

[30] Ix JH Sharma K. Mechanisms linking obesity, chronic kidney disease, and fatty liver disease: the roles of fetuin-A, adiponectin, and AMPK. J Am Soc Nephrol. (2010) 21:406–12. 10.1681/ASN.2009080820 20150538 PMC4473254

[31] Dixon JB Bhathal PS Hughes NR O'Brien PE. Nonalcoholic fatty liver disease: Improvement in liver histological analysis with weight loss. Hepatology (2004) 39:1647–54. 10.1002/hep.20251 15185306

[32] Scherer PE. Adipose tissue: from lipid storage compartment to endocrine organ. Diabetes (2006) 55:1537–45. 10.2337/db06-0263 16731815

[33] Yamauchi T Kamon J Ito Y Tsuchida A Yokomizo T Kita S. Cloning of adiponectin receptors that mediate antidiabetic metabolic effects. Nature (2003) 423:762–9. 10.1038/nature01705 12802337

[34] Yamauchi T Kamon J Minokoshi Y Ito Y Waki H Uchida S. Adiponectin stimulates glucose utilization and fatty-acid oxidation by activating AMP-activated protein kinase. Nat Med. (2002) 8:1288–95. 10.1038/nm788 12368907

[35] Asghari S Asghari-Jafarabadi M Somi MH Ghavami SM Rafraf M. Comparison of calorie-restricted diet and resveratrol supplementation on anthropometric indices, metabolic parameters, and serum sirtuin- 1 levels in patients with nonalcoholic fatty liver disease: a randomized controlled clinical trial. J Am Coll Nutr. (2018) 37:1–11. 10.1080/07315724.2017.1392264 29313746

[36] Jia L Li W Li J Li Y Song H Luan Y Lycium barbarum polysaccharide attenuates high-fat diet-induced hepatic steatosis by up-regulating SIRT (2016) 1 expression and deacetylase activity. Sci Rep. 6:36209 10.1038/srep36209 27824080 PMC5099939

[37] Fontana FY Colosio A De Roia GF Da Lozzo G Pogliaghi S. Anthropometrics of Italian senior male rugby union players: from elite to second division. Int J Sports Physiol Perform. (2015) 10:674–80. 10.1123/ijspp.2015-0014 25932593

[38] Zemski AJ Slater GJ Broad EM. Body composition characteristics of elite Australian rugby union athletes according to playing position and ethnicity. J Sports Sci. (2015) 33:970–8. 10.1080/02640414.2014.977937 25553727

[39] Jones B Till K Barlow M Lees M O'Hara JP Hind K. Anthropometric and three-compartment body composition differences between super league and championship rugby league players: considerations for the 2015 season and beyond. PLoS ONE (2015) 10:e0133188. 10.1371/journal.pone.0133188 26221720 PMC4519266

[40] Misu H Takayama H Saito Y Mita Y Kikuchi A Ishii KA. Deficiency of the hepatokine selenoprotein P increases responsiveness to exercise in mice through upregulation of reactive oxygen species and AMP-activated protein kinase in muscle. Nat Med. (2017) 23:508–16. 10.1038/nm.4295 28263310

[41] Kantartzis K Thamer C Peter A Machann J Schick F Schraml C. High cardiorespiratory fitness is an independent predictor of the reduction in liver fat during a lifestyle intervention in non-alcoholic fatty liver disease. Gut (2009) 58:1281–8. 10.1136/gut.2008.151977 19074179

[42] Kenneally S Sier JH Moore JB. Efficacy of dietary and physical activity intervention in non-alcoholic fatty liver disease: a systematic review. BMJ Open Gastroenterol. (2017) 4:e000139. 10.1136/bmjgast-2017-000139.eCollection2017 28761689 PMC5508801

[43] Orešič M Hyötyläinen T Kotronen A Gopalacharyulu P Nygren H Arola J. Prediction of non-alcoholic fatty-liver disease and liver fat content by serum molecular lipids. Diabetologia (2013) 56:2266–74. 10.1007/s00125-013-2981-2 23824212 PMC3764317

[44] Szczepaniak L.S Nurenberg P Leonard D Browning JD Reingold JS Grundy S. Magnetic resonance spectroscopy to measure hepatic triglyceride content: prevalence of hepatic steatosis in the general population. Am J Physiol Endocrinol Metab. (2005) 288:E462–8. 10.1152/ajpendo.00064.2004 15339742

[45] Williams CD Stengel J Asike MI Torres DM Shaw J Contreras M. Prevalence of nonalcoholic fatty liver disease and nonalcoholic steatohepatitis among a largely middle-aged population utilizing ultrasound and liver biopsy: a prospective study. Gastroenterology (2011) 140:124–31. 10.1053/j.gastro.2010.09.038 20858492

[46] Zelber-Sagi S Webb M Assy N Blendis L Yeshua H Leshno M. Comparison of fatty liver index with noninvasive methods for steatosis detection and quantification. World J Gastroenterol. (2013) 19:57–64. 10.3748/wjg.v19.i1.57 23326163 PMC3542754

[47] European Association for the Study of the Liver (EASL); European Association for the Study of Diabetes (EASD); European Association for the Study of Obesity (EASO) EASL-EASD-EASO Clinical Practice Guidelines for the management of non-alcoholic fatty liver disease. J Hepatol. (2016) 64:1388–402. 10.1016/j.jhep.2015.11.004 27062661

[48] Lee JH Kim D Kim HJ Lee CH Yang JI Kim W. Hepatic steatosis index: a simple screening tool reflecting nonalcoholic fatty liver disease. Dig Liver Dis. (2010) 42:503–8. 10.1016/j.dld.2009.08.002 19766548

[49] Lambert BS Oliver JM Katts GR Green JS Martin SE Crouse SF. DEXA or BMI: clinical considerations for evaluating obesity in collegiate division I-A American football athletes. Clin J Sport Med. (2012) 22:436–8. 10.1097/JSM.0b013e31825d5d65 22805182

